# Supplementary material for: Oxygen systems and quality of care for children with pneumonia, malaria and diarrhoea: Analysis of a stepped-wedge trial in Nigeria
Source: PLoS One. 2021 Jul 8;16(7):e0254229. doi: 10.1371/journal.pone.0254229 (PMC8266122; doi:10.1371/journal.pone.0254229)

## **S1 Fig. Characteristics of the Nigeria Oxygen Implementation Project stepped-wedge cluster randomised trial.**
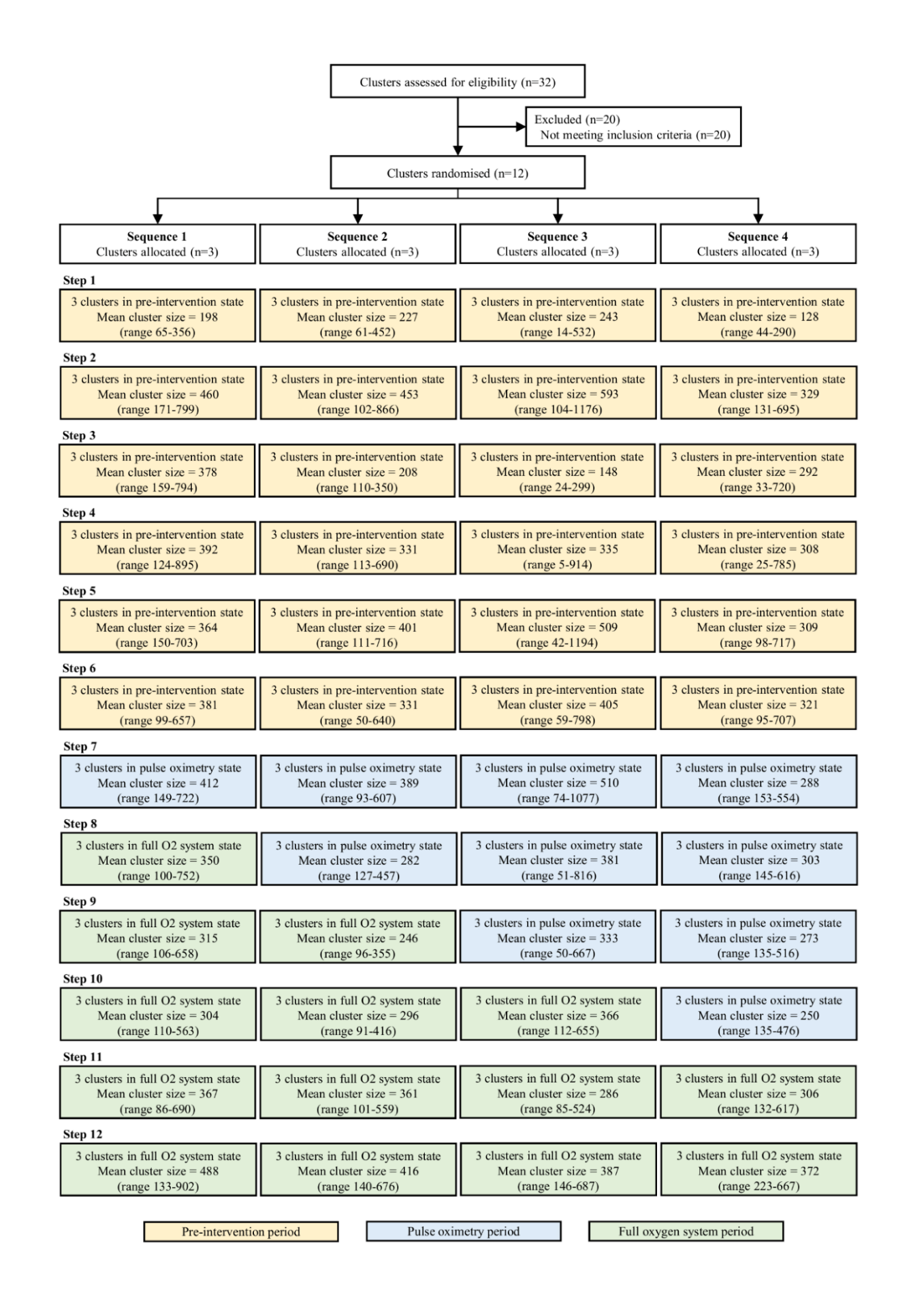

Supplement: S1 Fig — (DOCX) [file pone.0254229.s001.docx]
