# Supplementary material for: Oxygen systems and quality of care for children with pneumonia, malaria and diarrhoea: Analysis of a stepped-wedge trial in Nigeria
Source: PLoS One. 2021 Jul 8;16(7):e0254229. doi: 10.1371/journal.pone.0254229 (PMC8266122; doi:10.1371/journal.pone.0254229)
Supplement: S1 Table — (DOCX) [file pone.0254229.s003.docx]

## **S1 Table. Case definitions of severe pneumonia, severe malaria or diarrhoea with severe dehydration.**

| **Illness** | **Illness definition** | **Severe qualifying symptoms** |
| --- | --- | --- |
| **Pneumonia** | Cough or difficult breathing AND any of the following signs:   - Fast breathing (age 2-11 months, ≥50bpm; age 1-5 years, ≥40bpm) - Lower chest wall indrawing (respiratory distress) | Any of   - Severe respiratory distress (e.g. grunting, severe lower chest wall indrawing) - Central cyanosis (blue lips or tongue) - Altered conscious state (confusion, convulsions or coma) - Hypoxaemia (SpO2<90%) |
| **Malaria** | History of Fever/Temp >37.5 C AND Positive Malaria test (blood film or rapid diagnostic test) | Any of   - Altered conscious state (confusion, convulsions or coma) - Severe anaemia (packed cell volume <15%) |
| **Diarrhoea** | Watery Diarrhoea (>3 per day) that is not for >14 days duration | Any signs of severe dehydration (e.g. lethargy, sunken eyes, decreased skin turgor) |
