# Supplementary material for: Oxygen systems and quality of care for children with pneumonia, malaria and diarrhoea: Analysis of a stepped-wedge trial in Nigeria
Source: PLoS One. 2021 Jul 8;16(7):e0254229. doi: 10.1371/journal.pone.0254229 (PMC8266122; doi:10.1371/journal.pone.0254229)
Supplement: S2 Table — (DOCX) [file pone.0254229.s004.docx]

## **S2 Table.** **Quality of care scores for severe pneumonia, severe malaria and diarrhoea with severe dehydration.**

| Component | Score items | Modified score items (excluding pulse oximetry and oxygen therapy) | Points |
| --- | --- | --- | --- |
| Severe pneumonia | | | |
| Assessment | 1. All of temperature, heart rate, respiratory rate, and SpO_2_ documented on admission | All of temperature, heart rate and respiratory rate documented on admission | 1 |
|  | 1. Respiratory distress, feeding status and conscious state documented on admission | Respiratory distress, feeding status and conscious state documented on admission | 1 |
| Diagnosis | 1. Diagnosed with pneumonia on admission | Diagnosed with pneumonia on admission | 0.5 |
|  | 1. Classified correctly as severe pneumonia on admission | Classified correctly as severe pneumonia on admission | 0.5 |
| Treatment | 1. Prescribed appropriate antibiotic^1^ | Prescribed appropriate antibiotic^1^ | 1 |
|  | 1. Prescribed appropriate intravenous fluid therapy^2^ and prescribed appropriate oxygen therapy^4^ | Prescribed appropriate intravenous fluid therapy^2^ | 1 |
| Monitoring | 1. Vital signs documented at least three times per day and SpO_2_ documented at least twice per day if on oxygen | Vital signs documented at least three times per day | 1 |
| TOTAL |  |  | 6 |
| Severe malaria | | | |
| Assessment | 1. All of temperature, heart rate, respiratory rate, and SpO_2_ documented on admission | All of temperature, heart rate and respiratory rate documented on admission | 1 |
|  | 1. Blood sugar level, conscious state and feeding status documented on admission | Blood sugar level, conscious state and feeding status documented on admission | 1 |
| Diagnosis | 1. Diagnosed with malaria on admission | Diagnosed with malaria on admission | 0.5 |
|  | 1. Correct classification of malaria severity | Correct classification of malaria severity | 0.5 |
| Treatment | 1. Prescribed appropriate antimalarial treatment^1^ | Prescribed appropriate antimalarial treatment^1^ | 1 |
|  | 1. Prescribed appropriate intravenous fluid therapy^2^ and prescribed appropriate blood transfusion/supplement therapy^3^ and prescribed appropriate oxygen therapy^4^ | Prescribed appropriate intravenous fluid therapy^2^ and prescribed appropriate blood transfusion/supplement therapy^3^ | 1 |
| Monitoring | 1. Vital signs documented at least three times per day and SpO_2_ documented at least twice per day if on oxygen | Vital signs documented at least three times per day | 1 |
|  | **Diarrhoea with severe dehydration** | | |
| Assessment | 1. All of temperature, heart rate and respiratory rate documented on admission |  | 1 |
|  | 1. Weight, hydration, conscious state and feeding status documented on admission |  | 1 |
| Diagnosis | 1. Diagnosed with diarrhoea on admission |  | 0.5 |
|  | 1. Correct classification of severe dehydration |  | 0.5 |
| Treatment | 1. Prescribed appropriate intravenous fluid therapy^2^ |  | 1 |
|  | 1. Prescribed appropriate zinc and antibiotics^1^ |  | 1 |
| Monitoring | 1. Vital signs documented at least three times per day |  | 1 |

Notes: SpO_2_ = peripheral oxygen saturation, IM/IV = intramuscular or intravenous

Respiratory distress: gasping, grunting or severe chest indrawing

Feeding status: unable to breastfeed or drink adequately

Diagnoses as per case definitions (Appendix III)

1 = IV/IM Ampicillin/amoxicillin/Augmentin/Benzylpenicillin AND Gentamicin ± Azithromycin/clarithromycin/erythromycin for severe pneumonia; IV/IM Artesunate/Artemether OR IV quinine infusion for severe malaria; Antibiotics only given if: bloody diarrhoea (ciprofloxacin, ceftriaxone) OR prolonged diarrhoea ≥ 14 days (metronidazole) OR cholera - severe dehydration & ≥10 stool/day (ciprofloxacin, cotrimoxazole, trimethoprim and sulfamethoxazole, erythromycin) & must receive Zinc if age < five years and not persistent vomiting for severe diarrhoea

2 = Only given intravenous or nasogastric fluids if unable to continue oral feeding or severe dehydration AND appropriate choice of intravenous fluid: 0.9% NaCl ± 5% Dextrose/0.45% NaCl ± Dextrose/4.3% Dextrose/Saline/5% Dextrose Saline/5% Dextrose Water/Ringers’ Lactate/Half Strength Darrows/Full Strength Darrows/10% Dextrose water OR appropriate choice of nasogastric fluid: Only ReSoMal if malnutrition signs present AND rate not more than maintenance rate (4, 2, 1 rule) +20%, unless signs of shock, then up to bolus +20% accepted

3= If packed cell volume <15% must receive blood transfusion (volume not specified) OR Iron-folate supplement

4= Oxygen therapy given if: SpO_2_<90% OR SpO_2_ <95% and PCV<15% OR Coma/shock present
