# Supplementary material for: Oxygen systems and quality of care for children with pneumonia, malaria and diarrhoea: Analysis of a stepped-wedge trial in Nigeria
Source: PLoS One. 2021 Jul 8;16(7):e0254229. doi: 10.1371/journal.pone.0254229 (PMC8266122; doi:10.1371/journal.pone.0254229)
Supplement: S3 Table — (DOCX) [file pone.0254229.s005.docx]

# **S3 Tables. Quality of care score restricted and extended analysis.**

**Table 1. Effect of the intervention on quality of care score (including oxygen-related aspects of care), comparing the full oxygen system and pulse oximetry periods to baseline (extended analysis) and comparing full oxygen system period to pulse oximetry period (restricted analysis).**

| ­­ | **Severe pneumonia** | | | | **Severe malaria** | | | | **Diarrhoea with severe dehydration** | | | |
| --- | --- | --- | --- | --- | --- | --- | --- | --- | --- | --- | --- | --- |
|  | Extended^1^ | | Restricted^1^ | | Extended^1^ | | Restricted^2^ | | Extended^1^ | | Restricted^2^ | |
|  | aMD (95% CI) | *p*-value | aMD (95% CI) | *p*-value | aMD (95% CI) | *p-*value | aMD (95% CI) | *p*-value | aMD (95% CI) | *p*-value | aMD (95% CI) | *p*-value |
| **Score total ^3^** | | | | | | | | | | | | |
| **Pulse oximetry** | 0.66 (0.22, 1.09) | 0.003 | - | - | 1.26 (0.85, 1.68) | <0.001 | - | - | -0.29 (-0.78, 0.21) | 0.259 | - | - |
| **Full O2 system** | 1.39 (1.08, 1.69) | <0.001 | 0.65 (0.40, 0.89) | <0.001 | 1.53 (1.23, 1.83) | <0.001 | 0.32 (0.10, 0.54) | 0.004 | -0.12 (-0.46, 0.23) | 0.501 | 0.29 (-0.09, 0.67) | 0.135 |
| **Assessment component^3^** | | | | | | | | | | | | |
| **Pulse oximetry** | 0.41 (0.13, 0.69) | 0.004 | - | - | 0.43 (0.20, 0 .66) | <0.001 | - | - | -0.25 (-0.61, 0.10) | 0.163 | - | - |
| **Full O2 system** | 0.74 (0.54, 0.93) | <0.001 | 0.26 (0.11, 0.40) | <0.001 | 0.67 (0.51, 0.83) | <0.001 | 0.25  (0.11, 0.39) | 0.001 | -0.20 (-0.44, 0.04) | 0.097 | 0.05 (-0.20, 0.30) | 0.716 |
| **Diagnosis component^3^** | | | | | | | | | | | | |
| **Pulse oximetry** | -0.04 (-0.12, 0.04) | 0.342 | - | - | 0.15 (0.06, 0.25) | 0.002 | - | - | -0.03 (-0.17, 0.11) | 0.683 | - | - |
| **Full O2 system** | -0.02 (-0.08, 0.04) | 0.559 | 0.13 (-0.05, 0.07) | 0.664 | 0.12 (0.04, 0.10) | 0.002 | -0.04 ( -0.11, 0.24) | 0.217 | -0.05 (-0.15, 0.05) | 0.292 | 0.13 (-0.08, 0.10) | 0.791 |
| **Treatment component^3^** | | | | | | | | | | | | |
| **Pulse oximetry** | 0.15 (-0.013, 0.31) | 0.072 | - | - | 0.55 (0.33, 0.78) | <0.001 | - | - | -0.01 (-0.27, 0.25) | 0.942 | - | - |
| **Full O2 system** | 0.32 (0.20, 0.44) | <0.001 | 0.17 (0.06, 0.28) | 0.003 | 0.56 (0.39, 0.73) | <0.001 | 0.036 (-0.09, 0.17) | 0.55 | 0.10 (-0.08, 0.28) | 0.264 | 0.09 (-0.09, 0.26) | 0.327 |
| **Monitoring component^3^** | | | | | | | | | | | | |
| **Pulse oximetry** | 0.19 (0.001, 0.39) | 0.049 | - | - | 0.12 (-0.04, 0.27) | 0.139 | - | - | -0.11 (-0.34, 0.11) | 0.317 | - | - |
| **Full O2 system** | 0.37 (0.24, 0.51) | <0.001 | 0.17 (0.05, 0.29) | 0.004 | 0.18 (0.07, 0.29) | 0.002 | 0.07 ( -0.02, 0.17) | 0.141 | -0.002 (-0.15, 0.15) | 0.976 | 0.11(-0.05, 0.27) | 0.169 |

Acronyms: aMD = adjusted mean difference

1Extended analysis compares pulse oximetry and full oxygen system periods to the preintervention period

2 Restricted analysis compares full oxygen system and pulse oximetry periods.

3 Total = mean out of 6 total points, Assessment = total out of 2 points, Diagnosis = total out of 1 point, Treatment = total out of 2 points, Monitor = total out of 1 point

**Table 2. Effect of the intervention on modified quality of care scores (excluding oxygen-related aspects of care), comparing full oxygen system period to pulse oximetry period (restricted analysis) and comparing the full oxygen system and pulse oximetry periods to baseline (extended analysis).**

| ­­ | **Severe pneumonia** | | | | **Severe malaria** | | | |
| --- | --- | --- | --- | --- | --- | --- | --- | --- |
|  | Extended^1^ | | Restricted^2^ | | Extended^1^ | | Restricted^2^ | |
|  | aMD (95% CI) | *p*-value | aMD (95% CI) | *p*-value | aMD (95% CI) | *p-*value | aMD (95% CI) | *p*-value |
| **Modified total score^3^** | | | | | | | | |
| **Pulse oximetry** | -0.17 (-0.53, 0.19) | 0.351 | - | - | 0.68 (0.35, 1.00) | <0.001 | - | - |
| **Full O2 system** | 0.23 (-0.02, 0.48) | 0.072 | 0.38 (0.14, 0.61) | 0.002 | 0.65 (0.41, 0.89) | <0.001 | 0.01 (-0.16, 0.18) | 0.884 |
| **Modified assessment component^3^** | | | | | | | | |
| **Pulse oximetry** | -0.19 ( -0.44, 0.06) | 0.138 | - | - | -0.03 (-0.19, 0.13) | 0.705 | - | - |
| **Full O2 system** | -0.05 (-0.22, 0.13) | 0.601 | 0.13 (-0.01, 0.28) | 0.072 | -0.06 (-0.18, 0.06) | 0.303 | -0.02 (-0.11, 0.08) | 0.754 |
| **Modified treatment component^3^** | | | | | | | | |
| **Pulse oximetry** | 0.06 (-0.11, 0.22) | 0.511 | - | - | 0.47 (0.27, 0.68) | <0.001 | - | - |
| **Full O2 system** | 0.17 (0.05, 0.29) | 0.006 | 0.10 ( -0.01, 0.20) | 0.075 | 0.46 (0.31, 0.61) | <0.001 | 0.002 (-0.11, 0.12) | 0.975 |
| **Modified monitoring component^3^** | | | | | | | | |
| **Pulse oximetry** | 0.04 (-0.11, 0.20) | 0.601 | - | - | 0.07 (-0.06, 0.20) | 0.832 | - | - |
| **Full O2 system** | 0.13 (0.02, 0.24) | 0.016 | 0.09 (-0.02, 0.19) | 0.114 | 0.13 (0.04, 0.22) | 0.007 | 0.07 (-0.02, 0.15) | 0.131 |

Acronyms: aMD = adjusted mean difference

1Extended analysis compares pulse oximetry and full oxygen system periods to the preintervention period

2 Restricted analysis compares full oxygen system and pulse oximetry periods.

3 Total = mean out of 6 total points (includes non-modified diagnosis component), Assessment = total out of 2 points, Treatment = total out of 2 points, Monitor = total out of 1 point
