# Supplementary material for: Oxygen systems and quality of care for children with pneumonia, malaria and diarrhoea: Analysis of a stepped-wedge trial in Nigeria
Source: PLoS One. 2021 Jul 8;16(7):e0254229. doi: 10.1371/journal.pone.0254229 (PMC8266122; doi:10.1371/journal.pone.0254229)
Supplement: S4 Table — (DOCX) [file pone.0254229.s006.docx]

# **S4 Table. Population characteristics of participants, by study period and illness.**

|  | **Pre-intervention period** (n=3 128) | **Pulse oximetry only period** (n=1 564) | | **Full oxygen system period** (n=2 161) | **p-value^0^** |
| --- | --- | --- | --- | --- | --- |
|  | | | | | |
| **Infant (1-12 months)** |  |  | |  |  |
| - **Combined** | 876 (28.01%) | 473 (30.24%) | | 576 (26.65%) | p<0.001 |
| - **Severe pneumonia** | 417 (47.49%) | 239 (47.99%) | | 326 (49.85%) | p=0.020 |
| - **Severe malaria** | 304 (15.53%) | 142 (15.87%) | | 174 (12.91%) | p<0.001 |
| - **Diarrhoea with severe dehydration** | 155 (53.08%) | 92 (53.80%) | | 76 (47.80%) | p=0.394 |
| **Young child (1-5 years)** |  |  | |  |  |
| - **Combined** | 1 881 (60.13%) | 859 (54.92%) | | 1 183 (54.74%) | p<0.001 |
| - **Severe pneumonia** | 417 (47.49%) | 216 (43.37%) | | 274 (41.90%) | p=0.020 |
| - **Severe malaria** | 1,343 (68.59%) | 570 (63.69%) | | 839 (62.24%) | p=0.000 |
| - **Diarrhoea with severe dehydration** | 121 (41.44%) | 73 (42.69%) | | 70 (44.03%) | p=0.394 |
| **Older child (5-15 years)** |  |  | |  |  |
| - **Combined** | 371 (11.86%) | 232 (14.8%) | | 402 (18.6%) | p<0.001 |
| - **Severe pneumonia** | 44 (5.01%) | 43 (8.63%) | | 54 (8.26%) | p=0.020 |
| - **Severe malaria** | 311 (15.77%) | 183 (20.45%) | | 335 (24.85%) | p=0.000 |
| - **Diarrhoea with severe dehydration** | 16 (5.48%) | 6 (3.51%) | | 13 (8.18%) | p=0.394 |
|  | | | | | |
| **Age, months median (IQR)** |  |  | |  |  |
| - **Combined** | 21.0 (10.4-36) | 19.1 (10.0 – 39.0) | | 24.0 (11.0-48.0) | p<0.001^1^ |
| - **Severe pneumonia** | 12 (4.82-24) | 12 (5-24) | | 12 (4.09 – 24) | p=0.731^1^ |
| - **Severe malaria** | 26 (15-48) | 30 (15-48) | | 33.36 (18-59.43) | p<0.001^1^ |
| - **Diarrhoea with severe dehydration** | 11 (7.38-17.59) | 11 (7-16) | | 12 (8-18) | p=0.549^1^ |
| **Age, months mean (SD)** |  |  | |  |  |
| - **Combined** | 28.71 (26.90) | 30.05 (30.21) | | 34.16 (32.42) | p<0.001^1^ |
| - **Severe pneumonia** | 18.65 (22.39) | 20.52 (26.13) | | 20.98 (27.60) | p=0.1585^1^ |
| - **Severe malaria** | 34.83 (27.02) | 38.01 (31.01) | | 42.14 (32.46) | p<0.001^1^ |
| - **Diarrhoea with severe dehydration** | 17.93 (26.14) | 16.11 (23.33) | | 20.76 (28.16) | p=0.2627^1^ |
|  | | | | | |
| **Sex, % female** |  |  | |  |  |
| - **Combined** | 45.30% | 43.90% | | 44.70% | p=0.662 |
| - **Severe pneumonia** | 45.44% | 40.48% | | 48.02% | p=0.037 |
| - **Severe malaria** | 45.79% | 46.88% | | 42.75% | p=0.103 |
| - **Diarrhoea with severe dehydration** | 41.78% | 38.60% | | 47.50% | p=0.251 |
|  | | | | | |
| **Hospital type, % government** |  |  | |  |  |
| - **Combined** | 56.70% | 64.00% | | 56.50% | p<0.001 |
| - **Severe pneumonia** | 60.34% | 63.33% | | 57.60% | p=0.142 |
| - **Severe malaria** | 56.84% | 62.81% | | 56.94% | p=0.006 |
| - **Diarrhoea with severe dehydration** | 45.21% | 71.93% | | 48.12% | p=0.000 |
| **Length of Stay, days median (IQR)** |  |  | |  |  |
| - **Combined** | 4 (2-5) | 4 (2-5) | | 4 (2-6) | p=0.182^1^ |
| - **Severe pneumonia** | 4 (2-6) | 4 (2-6) | | 4 (2-7) | p=0.002^1^ |
| - **Severe malaria** | 4 (2-5) | 3 (2-5) | | 4 (2-5) | p=0.873^1^ |
| - **Diarrhoea with severe dehydration** | 4 (2-5) | 4 (3-5) | | 4 (2-5) | p=0.916^1^ |
|  | | | | | |
| **Child diagnoses and presenting signs^2^** | | | | |  |
| **Severe Pneumonia** | 1039 (33.0%) | | 571 (36.4%) | 774 (35.5%) | p=0.038 |
| **Severe Malaria** | 2005 (63.7%) | | 921 (58.7%) | 1378 (63.2%) | p=0.002 |
| **Severe Diarrhoea** | 292 (9.3%) | | 171 (10.9%) | 160 (7.3%) | p=0.001 |
|  | | | | |  |
| **Malnutrition** |  | |  |  |  |
| - **Combined** | 56 (1.8%) | | 43 (2.8%) | 42 (1.9%) | p=0.080 |
| - **Severe pneumonia** | 17 (1.95%) | | 12 (2.43%) | 16 (2.45%) | p=0.752 |
| - **Severe malaria** | 26 (1.33%) | | 17 (1.9%) | 17 (1.25%) | p=0.394 |
| - **Diarrhoea with severe dehydration** | 13 (4.45%) | | 14 (8.19%) | 9 (5.62%) | p=0.250 |
| **HIV** |  | |  |  |  |
| - **Combined** | 2 (0.06%) | | 5 (0.32%) | 4 (0.18%) | p=0.11^3^ |
| - **Severe pneumonia** | 1 (0.11%) | | 2 (0.41%) | 2 (0.31%) | p=0.535^3^ |
| - **Severe malaria** | 1 (0.05%) | | 2 (0.22%) | 1 (0.07%) | p=0.349^3^ |
| - **Diarrhoea with severe dehydration** | 0 (0.00%) | | 1 (0.58%) | 1 (0.62%) | p=0.282^3^ |
| **Severe respiratory distress** |  | |  |  |  |
| - **Combined** | 851 (27.1%) | | 455 (29.0%) | 523 (24.0%) | p=0.002 |
| - **Severe pneumonia** | 657 (74.66%) | | 344 (68.94%) | 392 (58.57%) | p=0.000 |
| - **Severe malaria** | 177 (8.97%) | | 90 (10.02%) | 119 (8.74%) | p=0.557 |
| - **Diarrhoea with severe dehydration** | 17 (5.82%) | | 12 (12.28%) | 12 (7.50%) | p=0.046 |
| **Packed Cell Volume below 15%** |  | |  |  |  |
| - **Combined** | 672 (23.0%) | | 273 (18.7%) | 508 (24.8%) | p<0.001 |
| - **Severe pneumonia** | 40 (5.45%) | | 22 (5.16%) | 23 (4.04%) | p=0.484 |
| - **Severe malaria** | 629 (32.52) | | 249 (28.06%) | 482 (36.05%) | p=0.000 |
| - **Diarrhoea with severe dehydration** | 3 (1.2%) | | 2 (1.34%) | 3 (2.17%) | p=0.741 |
| **Signs of Severe dehydration** |  | |  |  |  |
| - **Combined** | 322 (10.2%) | | 194 (12.4%) | 183 (8.4%) | p<0.001 |
| - **Severe pneumonia** | 8 (0.91%) | | 11 (2.20%) | 7 (1.06%) | p=0.101 |
| - **Severe malaria** | 22 (1.11%) | | 12 (1.34%) | 16 (1.18%) | p=0.878 |
| - **Diarrhoea with severe dehydration** | 292 (100%) | | 171 (100%) | 160 (100%) | - |
| **Unable to feed** |  | |  |  |  |
| - **Combined** | 791 (25.1%) | | 371 (23.7%) | 551 (25.3%) | p=0.460 |
| - **Severe pneumonia** | 264 (30.00%) | | 130 (26.05%) | 168 (25.53%) | p=0.103 |
| - **Severe malaria** | 453 (22.95%) | | 205 (22.83%) | 328 (24.10%) | p=0.692 |
| - **Diarrhoea with severe dehydration** | 74 (25.34%) | | 36 (21.05%) | 55 (34.38%) | p=0.019 |
| **Hypoxaemia (SpO2<90%)^4^** |  | |  |  |  |
| - **Combined** | 32 (14.2%) | | 228 (23.0%) | 497 (24%) | p=0.004 |
| - **Severe pneumonia** | 13 (1.48%) | | 142 (28.46%) | 298 (45.29%) | p<0.000 |
| - **Severe malaria** | 17 (0.86%) | | 75 (8.35%) | 183 (13.45%) | p<0.000 |
| - **Diarrhoea with severe dehydration** | 2 (0.68%) | | 11 (6.34%) | 16 (10.00%) | p<0.000 |
| **Severe Hypoxaemia (SpO_2_<80%)^4^** |  | |  |  |  |
| - **Combined** | 15 (6.7%) | | 90 (9.1%) | 240 (11.6%) | p=0.016 |
| - **Severe pneumonia** | 4 (0.45%) | | 54 (10.82%) | 143 (21.73%) | p<0.000 |
| - **Severe malaria** | 10 (0.51%) | | 31 (3.45%) | 86 (6.32%) | p<0.000 |
| - **Diarrhoea with severe dehydration** | 1 (0.34%) | | 5 (2.92%) | 11 (6.88%) | p<0.000 |
| **Central cyanosis** |  | |  |  |  |
| - **Combined** | 18 (0.57%) | | 14 (0.89%) | 40 (1.84) | p<0.001 |
| - **Severe pneumonia** | 12 (1.36%) | | 7 (1.4%) | 8 (1.22%) | p=0.955 |
| - **Severe malaria** | 4 (0.20%) | | 4 (0.45%) | 30 (2.20%) | p<0.000 |
| - **Diarrhoea with severe dehydration** | 2 (0.68%) | | 3 (1.75%) | 2 (1.25%) | p=0.565 |
| **Signs of shock** |  | |  |  |  |
| - **Combined** | 15 (0.48%) | | 12 (0.77%) | 15 (0.69%) | p=0.413 |
| - **Severe pneumonia** | 4 (0.45%) | | 1 (0.20%) | 4 (0.61%) | p=0.584 |
| - **Severe malaria** | 3 (0.15%) | | 7 (0.78%) | 8 (0.59%) | p= 0.030 |
| - **Diarrhoea with severe dehydration** | 8 (2.74%) | | 4 (2.34%) | 3 (1.88%) | p= 0.846 |
| **Convulsions** |  | |  |  |  |
| - **Combined** | 1194 (50.5%) | | 499 (46.5%) | 696 (49.9%) | p=0.085 |
| - **Severe pneumonia** | 93 (17.29%) | | 34 (12.98%) | 53 (16.51%) | p=0.287 |
| - **Severe malaria** | 1,087 (65.96%) | | 456 (63.43%) | 631 (63.74%) | p=0.358 |
| - **Diarrhoea with severe dehydration** | 14 (7.91%) | | 9 (9.78%) | 12 (14.29%) | p=0.273 |
| **Confusion or lethargy** |  | |  |  |  |
| - **Combined** | 713 (35.3%) | | 470 (49.2%) | 436 (35.2%) | p<0.001 |
| - **Severe pneumonia** | 95 (17.43%) | | 55 (20.15%) | 53 (15.19%) | p=0.269 |
| - **Severe malaria** | 534 (41.95%) | | 339 (60.75%) | 342 (43.73%) | p<0.001 |
| - **Diarrhoea with severe dehydration** | 84 (41.38%) | | 76 (61.29%) | 41 (37.61%) | p<0.001 |
| **Coma or barely conscious** |  | |  |  |  |
| - **Combined** | 403 (12.8%) | | 183 (11.7%) | 408 (18.7%) | p<0.001 |
| - **Severe pneumonia** | 44 (5.00%) | | 20 (4.01%) | 46 (6.99%) | p=0.066 |
| - **Severe malaria** | 346 (17.53%) | | 158 (17.59%) | 346 (25.42%) | p<0.001 |
| - **Diarrhoea with severe dehydration** | 13 (4.45%) | | 5 (2.92%) | 16 (10.00%) | p=0.011 |
| **Any WHO Emergency Sign** |  | |  |  |  |
| - **Combined** | 2605 (82.8%) | | 1371 (87.4%) | 1860 (85.7%) | p<0.001 |
| - **Severe pneumonia** | 766 (87.05%) | | 448 (89.78%) | 606 (92.10%) | p=0.006 |
| - **Severe malaria** | 1,574 (79.74%) | | 764 (85.08%) | 1,108 (81.41%) | p=0.003 |
| - **Diarrhoea with severe dehydration** | 265 (90.75%) | | 159 (92.98%) | 146 (91.25%) | p=0.703 |

Data are *n* (%) unless otherwise indicated. Severe pneumonia, severe malaria and diarrhoea with severe dehydration as per our case definitions. Other diagnoses as per admission diagnosis.
Abbreviations: SD = standard deviation, IQR = interquartile range, SpO2 = peripheral oxygen saturation

^0^Pearson Chi^2^ unless otherwise specified

^1^K-sample equality of medians test and Wilks’ lambda test of means

^2^Multiple diagnoses permitted

^3^ Fisher's exact test

^4^Limited hypoxaemia data available in preintervention period, mostly from a single hospital.

.
