# Supplementary material for: Oxygen systems and quality of care for children with pneumonia, malaria and diarrhoea: Analysis of a stepped-wedge trial in Nigeria
Source: PLoS One. 2021 Jul 8;16(7):e0254229. doi: 10.1371/journal.pone.0254229 (PMC8266122; doi:10.1371/journal.pone.0254229)
Supplement: S5 Table — (DOCX) [file pone.0254229.s007.docx]

# **S5 Tables. Quality of care score feature and component sub-analysis.**

**Table 1. Proportion of children with severe pneumonia who received care/had care performed by each feature of QOC score component**

| **Score feature** | **Severe pneumonia** | | |
| --- | --- | --- | --- |
|  | **Pre-study**  **(n=1039)** | **Pulse oximetry only**  **(n=571)** | **Full oxygen period**  **(n=774)** |
| **Assessment Features** | | | |
| Documented temperature on admission | 973 (93.65%) | 539 (94.40%) | 748 (96.64%) |
| Documented heart rate on admission | 995 (95.77%) | 561 (98.25%) | 769 (99.35%) |
| Documented respiratory rate on admission | 1,022 (98.36%) | 566 (99.12%) | 766 (98.97%) |
| Documented SpO_2_ on admission | 59 (5.68%) | 433 (75.83%) | 737 (95.22%) |
| Respiratory distress documented on admission | 872 (83.93%) | 444 (77.76%) | 584 (75.45%) |
| Feeding status documented on admission | 557 (53.61%) | 275 (48.16%) | 349 (45.09%) |
| Conscious state documented on admission | 810 (77.96%) | 449 (78.63%) | 635 (82.04%) |
| **Diagnosis features** | | | |
| Diagnosed with pneumonia on admission | 661 (63.62%) | 339 (59.37%) | 449 (58.01%) |
| Classified correctly as severe pneumonia on admission | 5 (35.71%) | 4 (28.57%) | 5 (35.71%) |
| **Treatment features** | | | |
| Prescribed appropriate antibiotic^1^ | 52 (5.00%) | 22 (3.85%) | 45 (5.81%) |
| Prescribed appropriate intravenous fluid therapy^2^ | 666 (64.10%) | 398 (69.70%) | 523 (67.57%) |
| Prescribed appropriate oxygen therapy, (n= those indicated)^4^ | 446/889 (50.17%) | 155/291 (76.18%) | 334/404 (82.67%) |
| **Monitoring features** | | | |
| Vital signs documented at least three times per day | 868 (83.54%) | 465 (81.44%) | 696 (89.92%) |
| SpO_2_ documented at least twice per day if on oxygen | 21/535 (3.93%) | 108/226 (47.79%) | 388/481 (80.67%) |

Note: of children who did not receive appropriate antibiotics for severe pneumonia during all periods (n=2,265), 198 (8.74%) received no antibiotics, 223 (9.85%) received oral antibiotics only, 837 (36.95%) received IV ceftriaxone and 910 (40.18%) received IV cefuroxime.

**Table 2. Proportion of children with severe malaria who received care/had care performed by each feature of QOC score component.**

| **Score feature** | **Severe malaria** | | |
| --- | --- | --- | --- |
|  | **Pre-study**  **(n=2,005)** | **Pulse oximetry only**  **(n=921)** | **Full oxygen period**  **(n=1,378)** |
| **Assessment features** | | | |
| Documented temperature on admission | 1,931 (96.31%) | 898 (97.50%) | 1,346 (97.68%) |
| Documented heart rate on admission | 1,892 (94.36%) | 904 (98.15%) | 1,368 (99.27%) |
| Documented respiratory rate on admission | 1,851 (92.32%) | 890 (96.63%) | 1,356 (98.40%) |
| Documented SpO_2_ on admission | 167 (8.33%) | 539 (58.52%) | 1,315 (95.43%) |
| Blood Sugar Levels documented on admission | 580 (45.63%) | 283 (43.34%) | 431 (39.61%) |
| Feeding status documented on admission | 1,032 (51.47%) | 414 (44.95%) | 515 (37.37%) |
| Conscious state documented on admission | 1,656 (82.59%) | 821 (89.14%) | 1,256 (91.15%) |
| **Diagnosis features** | | | |
| Diagnosed with malaria on admission | 1,693 (84.44%) | 789 (85.67%) | 1,145 (83.09%) |
| Classified correctly as severe malaria on admission | 1,253 (63.06%) | 571 (62.27%) | 906 (65.89%) |
| **Treatment features** | | | |
| Prescribed appropriate antimalarial^1^ | 1,290 (64.34%) | 757 (82.19%) | 1,200 (87.08%) |
| Prescribed appropriate intravenous fluid therapy^2^ | 1,360 (67.83%) | 621 (67.43%) | 980 (71.12%) |
| Prescribed appropriate blood transfusion/supplement therapy^3^ | 1,984 (98.95%) | 916 (99.46%) | 1,364 (98.98%) |
| Prescribed appropriate oxygen therapy (n= those indicated)^4^ | 148/957 (15.45%) | 78/366 (21.31%) | 231 (46.29%) |
| **Monitoring features** | | | |
| Vital signs documented at least three times per day | 1,688 (84.19%) | 760 (82.52%) | 1,224 (88.82%) |
| SpO_2_ documented at least twice per day if on oxygen | 25/273 (9.16%) | 60 (53.10%) | 256 (66.49%) |

**Table 3. Proportion of children with diarrhoea with severe dehydration who received care/had care performed by each feature of QOC score component**

| **Score feature** | **Diarrhoea with severe dehydration** | | |
| --- | --- | --- | --- |
|  | **Pre-study**  **(n=292)** | **Pulse oximetry only**  **(n=171)** | **Full oxygen period**  **(n=160)** |
| **Assessment features** | | | |
| Documented temperature on admission | 272 (93.15%) | 162 (94.74%) | 153 (95.62%) |
| Documented heart rate on admission | 273 (93.49%) | 166 (97.08%) | 159 (99.38%) |
| Documented respiratory rate on admission | 272 (93.15%) | 161 (94.15%) | 158 (98.75%) |
| Weight documented on admission | 254 (86.99%) | 145 (84.80%) | 134 (83.75%) |
| Hydration status documented on admission | 270 (92.47%) | 161 (94.15%) | 160 (100.00%) |
| Feeding status documented on admission | 152 (52.05%) | 69 (40.35%) | 83 (51.88%) |
| Conscious state on admission | 231 (79.11%) | 150 (87.72%) | 143 (89.38%) |
| **Diagnosis features** | | | |
| Diagnosed with diarrhoea on admission | 255 (87.33%) | 137 (80.12%) | 136 (85.00%) |
| Classified correctly as severe dehydration on admission | 250 (86.21%) | 158 (92.40%) | 150 (93.75%) |
| **Treatment features** | | | |
| Prescribed appropriate IV fluid therapy | 34 (11.64%) | 17 (9.94%) | 23 (14.37%) |
| Prescribed appropriate zinc replacement | 259 (88.70%) | 159 (92.98%) | 146 (91.25%) |
| Prescribed/withheld appropriate antibiotic therapy | 52 (17.81%) | 21 (12.28) | 32 (20.00%) |
| **Monitoring features** | | | |
| Vital signs documented at least three times per day | 242 (82.88%) | 133 (77.78%) | 141 (88.12%) |

Notes: SpO_2_ = peripheral oxygen saturation, IM/IV = intramuscular or intravenous

Respiratory distress: gasping, grunting or severe chest indrawing

Feeding status: unable to breastfeed or drink adequately

Diagnoses as per case definitions (Appendix III)

1 = IV/IM Ampicillin/amoxicillin/Augmentin/benzylpenicillin AND gentamicin ± azithromycin/clarithromycin/erythromycin for severe pneumonia; IV/IM Artesunate/Artemether OR IV quinine infusion for severe malaria; Antibiotics only given if: bloody diarrhoea (ciprofloxacin, ceftriaxone) OR prolonged diarrhoea ≥ 14 days (metronidazole) OR cholera - severe dehydration & ≥10 stool/day (ciprofloxacin, cotrimoxazole, trimethoprim and sulfamethoxazole, erythromycin) & must receive Zinc if age < five years and not persistent vomiting for severe diarrhoea

2 = Only given intravenous or nasogastric fluids if unable to continue oral feeding or severe dehydration AND appropriate choice of intravenous fluid: 0.9% NaCl ± 5% Dextrose/0.45% NaCl ± Dextrose/4.3% Dextrose/Saline/5% Dextrose Saline/5% Dextrose Water/Ringers’ Lactate/Half Strength Darrows/Full Strength Darrows/10% Dextrose water OR appropriate choice of nasogastric fluid: Only ReSoMal if malnutrition signs present AND rate not more than maintenance rate (4, 2, 1 rule) +20%, unless signs of shock, then up to bolus +20% accepted

3= If packed cell volume <15% must receive blood transfusion (volume not specified) OR Iron-folate supplement

4= Oxygen therapy given if: SpO_2_<90% OR SpO_2_ <95% and Packed Cell Volume <15% OR coma/shock present
